# Optimal configurations for stiffness and compliance in human & robot arms

**DOI:** 10.1371/journal.pone.0302987

**Published:** 2024-05-29

**Authors:** Jon Woolfrey, Arash Ajoudani, Wenjie Lu, Lorenzo Natale

**Affiliations:** 1 School of Electronic & Electrical Engineering, University of Leeds, Woodhouse, United Kingdom; 2 Center for Intelligent & Robotic Systems, Istituto Italiano di Tecnologia, Genoa, GE, Italy; 3 School of Mechatronics & Automation, Harbin Institute of Technology, Shenzen, Guangdong, China; University rehabilitation institute, SLOVENIA

## Abstract

Research in neurophysiology has shown that humans are able to adapt the mechanical stiffness at the hand in order to resist disturbances. This has served as inspiration for optimising stiffness in robot arms during manipulation tasks. Endpoint stiffness is modelled in Cartesian space, as though the hand were in independent rigid body. But an arm is a series of rigid bodies connected by articulated joints. The contribution of the joints and arm configuration to the endpoint stiffness has not yet been quantified. In this paper we use mathematical optimisation to find conditions for maximum stiffness and compliance with respect to an externally applied force. By doing so, we can retroactively explain observations made about humans using these mathematically optimal conditions. We then show how this optimisation can be applied to robotic task planning and control. Experiments on a humanoid robot show similar arm posture to that observed in humans. This suggests there is an underlying physical principle by which humans optimise stiffness. We can use this to derive natural control methods for robots.

## Introduction

Research in human physiology has shown that humans are able to modify the mechanical stiffness of their arms to improve task performance [1, 2]. This is achieved by leveraging musculoskeletal properties through a combination of muscle contraction and arm configuration [2, 3]. Naturally, robotics researchers have attempted to emulate this behaviour in autonomous manipulation tasks. The Cartesian stiffness ellipsoid was shown to be a good predictor in humans [4], and serves as a basis for developing robotic control methods. However, an arm is a series of rigid bodies connected by articulated joints. The joints contribute to stiffness at the hand has not been adequately explained. By analysing the Cartesian stiffness as a function of the joints, it can provide better insight in to how humans and robots can regulate interaction with external forces. Moreover, it is possible to embed robots—particularly humanoids—with more natural control behaviours.

For a single rigid body, Hooke’s law states that its deflection is linearly proportional to an applied force [5] governed by the equation:

$$\begin{array}{c} \text{w}={\text{w}}_{0}+\delta \text{w}={\text{K}}_{c}\delta \text{x} \end{array}$$ (1)

where:

-
$\text{w}\in {\mathbb{R}}^{\text{m}}$ is a wrench of forces (N) and moments (Nm),
-
${\text{K}}_{\text{c}}={\text{K}}_{\text{c}}^{\text{T}}\in R^{m\times m}$ is the Cartesian stiffness matrix,
-
$\text{x}\in R^{\text{m}}$ is the position and orientation (pose) of said rigid body, and
- *δ* denotes an infinitesimally small change from some nominal state.

The nominal wrench is assumed to be **w**_0_ = **0** when no external force is applied. As aforementioned, a human or robot arm is composed of many rigid bodies connected in series. The displacement of the endpoint is due to the displacement of the joints and ought to be accounted for when analysing stiffness. The pose for the endpoint of a mechanism is computed from the joint angles $\text{q}\in R^{n}$ known as forward kinematics:

$$\begin{array}{c} \text{x}=\text{g}(\text{q}). \end{array}$$ (2)

For an infinitesimally small displacement the relationship between the endpoint deflection and joint deflection is given by:

$$\begin{array}{c} \delta \text{x}=\text{J}(\text{q})\delta \text{q} \end{array}$$ (3)

where $\text{J}=\partial \text{g}/\partial \text{q}\in R^{6\times n}$ is the Jacobian matrix. To find the relationship between endpoint force and joint torques $\tau \in R^{\text{n}}$ we can equate the power between the joint space and Cartesian space to show that:

$$\begin{array}{c} {\dot{\text{q}}}^{\text{T}}\tau ={\dot{\text{x}}}^{\text{T}}\text{w}={\dot{\text{q}}}^{\text{T}}{\text{J}}^{\text{T}}\text{w} \end{array}$$ (4a)

$$\begin{array}{c} ∴\tau =J^{\text{T}}\text{w}. \end{array}$$ (4b)

We can use this relationship to analyse how the kinematics affects the ability of a human or robot to produce forces at the endpoint. If we assume isotropic joint torque:

$$\begin{array}{c} \tau^{\text{T}}\tau =1 \end{array}$$ (5a)

then by substituting Eq 1 in to Eq 4b, Eq 4b in to Eq 5a we obtain:

$$\begin{array}{c} {\text{w}}^{\text{T}}{\text{JJ}}^{\text{T}}\text{w}=1 \end{array}$$ (5b)

$$\begin{array}{c} \delta {\text{x}}^{\text{T}}{\text{K}}_{\text{c}}{\text{JJ}}^{\text{T}}{\text{K}}_{\text{c}}\delta \text{x}=1. \end{array}$$ (5c)

Eq 5a is the equation for an n-dimensional sphere in the joint space, centered at zero. Eq 5b denotes the Cartesian force ellipsoid, and Eq 5c the Cartesian stiffness ellipsoid. The eigenvectors of the matrix **K**_c_**JJ**^T^**K**_c_ correspond to the principle radii of the stiffness ellipsoid. They have been shown to be a good predictor of stability in the human arm when subject to external forces [4]. Notably, the stiffness ellipsoid is a function of the Jacobian **J**(**q**) which is a function of the joint positions **q**. Therefore, changing the joint configuration can maximise or minimise the stiffness ellipsoid in different directions.

To further illustrate this, we can examine the joint stiffness as opposed to the Cartesian stiffness. Eq 1 implies that **K**_c_ = ∂**w**/∂**x**. Similarly, we can evaluate the joint stiffness matrix using the derivative product rule, substituting in Eq 4b, to obtain:

$$\begin{array}{c} {\text{K}}_{\text{q}}≜\frac{\partial \tau }{\partial \text{q}}=\underset{{\text{K}}_{\text{a}}}{\underset{︸}{{\text{J}}^{\text{T}}{\text{K}}_{\text{c}}\text{J}}}+\underset{{\text{K}}_{\text{p}}}{\underset{︸}{\frac{\partial {\text{J}}^{\text{T}}}{\partial \text{q}}{\text{K}}_{\text{c}}\delta \text{x}}}. \end{array}$$ (6)

The first term on the right hand side ${\text{K}}_{\text{a}}\in R^{\text{n}\times \text{n}}$ is the active stiffness matrix. It pertains to the joint torques that attempt to restore the endpoint to its nominal pose when displaced. It is sometimes referred to as the common mode stiffness (CMS) in the robotics literature [6–9]. The second term ${\text{K}}_{\text{p}}\in R^{\text{n}\times \text{n}}$ is the passive stiffness which results from a change in configuration. In robotics it has been referred to as the configuration-dependent stiffness (CDS) [6–9].

In humans, active stiffness is achieved through muscle contraction. Although, literature suggests that it only modifies the volume of the stiffness ellipsoid rather than the shape [2]. In fact, under fixed arm configurations, it appears that humans have little control over the direction of stiffness [10]. This implies that active stiffness **K**_a_ is subordinate to passive stiffness **K**_p_, and that changing arm configuration is a better strategy than tensing one’s muscles. Several other studies support this notion. For example, it was observed that muscle contractions change with arm configuration [3] hinting at its significance in shaping stiffness. Other research directly hypothesized that resisting displacement of the hand is a combination of joint torques (i.e. muscle contraction) and joint angles [4]. The decomposition of the joint stiffness matrix Eq 6 makes this evident.

The significance of arm posture, and hence **K**_p_, had been hypothesized as far back as 1985 [11]. This research concluded that it was the *primary* control input of the central nervous system. The general consensus in the neurophysiology literature appears be that increased mechanical advantage can supplant the need for muscle contraction [1, 2, 4, 12]. The latter is metabolically costly, and therefore ought to be minimised where possible. When permitted, humans will naturally choose arm configurations that increase the stiffness in the direction of disturbances at the hand [12]. The authors hypothesized that kinematic redundancy may be exploited to regulate stiffness. Many descriptions have been written about the measured stiffness and observed arm configuration, but this relationship has not been quantified.

Control of robot arms had traditionally been concerned with accurate tracking of a desired joint position or Cartesian pose. Driven by industry, there was a need for fast and precise manipulation. However, this precision necessitates high stiffness. This can lead to dangerously high contact forces if the robot is physically disturbed. Conversely, impedance control was proposed as a method for regulating the interaction forces between robots and their environment [13–15] (Ironically, these series of papers were the catalyst for studying human stiffness regulation in neurophysiology). This method has since become a staple for robots operating in unstructured environments or interacting with humans. By regulating contact forces, robots can be made compliant to disturbances and uncertainty. We can impose the following second-order differential equation on the endpoint dynamics [16]:

$$\begin{array}{c} \text{w}=\Lambda \underset{\ddot{\text{e}}}{\underset{︸}{\left({\ddot{\text{x}}}_{\text{d}}-\ddot{\text{x}}\right)}}+\text{D}\underset{\dot{\text{e}}}{\underset{︸}{\left({\dot{\text{x}}}_{\text{d}}-\dot{\text{x}}\right)}}+\text{K}\underset{\text{e}}{\underset{︸}{\left({\text{x}}_{\text{d}}-\text{x}\right)}} \end{array}$$ (7)

which is analogous to a mass-spring-damper where:

-
${\text{x}}_{\text{d}}\in R^{6}$ is some desired pose,
-
$\Lambda \in R^{6\times 6}$ is the Cartesian mass-inertia matrix,
-
$\text{K}\in R^{6\times 6}$ is a damping matrix, and
-
$\text{K}\in R^{6\times 6}$ a stiffness matrix, and
-
$\dot{\text{x}}=\frac{\text{d}\text{x}}{\text{dt}}$ is the time derivative.

The inertia **Λ**, damping **D**, and stiffness **K** can be designed to produce a desired dynamic response. For instance, the inertia can be reduced to make it easier for a human to manipulate the robot. Or, a low stiffness may be applied so the robot complies to unexpected disturbances.

There is a natural affinity here between the position error feedback **Ke** and the Cartesian stiffness modelling Eq 1. In fact, Hogan asserted that active feedback was not the best method compared to “exploiting the intrinsic properties of mechanical hardware” [13]. This was demonstrated in the effects of a robotic polishing task [17]. By changing the joint configuration of a robot, the natural compliance in its structure was shown to improve surface finish. Despite this, the robotics community has still not adopted control of the passive stiffness as standard practice. For example, 31 years after Hogan had published his research, a method of compliance optimisation was developed for a robotic handover task [18]. The passive stiffness term **K**_p_ was assumed to be negligible, thus the joint stiffness could be conveniently solved using only **K**_a_. In 1994, research showed that reconfiguring a robot arm could reduce impact forces on the endpoint [19]. But 24 years later active force-feedback was proposed for floating manipulation without considering passive compliance of the robot configuration itself [20].

As surmised by [12], kinematic redundancy may be exploited to enhance performance depending on the task. This has been actively applied in robotics. Inspired by the fact that humans can find “natural” postures for a given task, a method for reorienting the Cartesian stiffness ellipsoid was proposed by [21]. This relied on correcting the error between the desired and actual stiffness. It did not actively exploit redundancy. Nevertheless, a simulated robot arm was shown to reconfigure itself and reshape the Cartesian stiffness ellipsoid accordingly. Later, kinematic redundancy was directly utilized using this stiffness error method [6, 22]. Only the active stiffness component **K**_a_ was considered, and was assumed to be constant. This further omits configuration dependency since **K**_a_ is itself a function of the joint positions. This method was later applied to a two-handed manipulation task [7]. The role of passive stiffness **K**_p_ in impedance control was finally considered for a redundant robot in [8, 23]. The control problem optimised the Cartesian stiffness ellipsoid to reconfigure the arm whilst keeping the endpoint stationary.

Inspired by neurophysiology, the robotics researchers have demonstrated that they can similarly shape the Cartesian stiffness ellipsoid in robotic arms. They concluded—inductively—that since the resulting robot arm configurations look “natural” then the control methods are “natural” [6, 7, 21]. “Natural” is not defined. The neurophysiology literature only makes qualitative remarks regarding measured stiffness and observed arm posture in this regard.

The purpose of this paper is to quantify how the joint configuration of a serial link mechanism contributes to stiffness and compliance at its endpoint. Specifically, Cartesian stiffness is treated as a mathematical optimisation problem. We then derive the conditions for the minima (optimal stiffness) and maxima (optimal compliance) with respect to how the joint axes align with a wrench at the endpoint. A similar treatment has been given to parallel mechanisms [24], although we consider both stiffness and compliance (its inverse).

In doing so, three important contributions are made:

1. Observations of humans in the neurophysiology literature can be retroactively explained using these optimality conditions,
2. The optimisation function can be used as a metric for robotic task planning, and
3. Applying the optimisation to a humanoid robot leads to arm configurations similar to those observed in humans.

Roboticists have frequently looked to biological principles to derive control methods. For example, research in neurophysiology suggests human arm motion follows a minimum-jerk trajectory [25]. This type of trajectory generation has been implemented as standard in the humanoid iCub robot [26]. Motion tracking has also been used to replicate human-like movement in androids [27]. More recently, learning from human demonstration has been used for trajectory generation in whole-body control of humanoid robots [28]. Illuminating the role of arm configuration in Cartesian stiffness can enhance our understanding of human behaviour and stiffness control methods in autonomous systems. For instance, a method for estimating Cartesian stiffness in human arms was developed in [29], which was later used to emulate similar behaviour in robots [30]. Although, said method relied on the fact that the human arm can be reduced to 2 links, and does not consider rotational stiffness. The results of this paper generalise to mechanisms with any number of links and joints, both revolute and prismatic, and considers translation and rotational stiffness.

Another minor contribution is to show that maximising stiffness at the hand simultaneously reduces the joint torques required to resist external forces. It had been speculated in neurophysiology that stiffness optimisation is connected to energy reduction. Interestingly, some robotics researchers had developed a control method for reducing joint torques from external loads [31]. They noted the joint stiffness matrix appeared when taking the derivative of the torque minimization function. We show that zero joint torque is a condition for maximum stiffness. For a robotic arm, the Cartesian stiffness can be arbitrarily shaped through an appropriate control design. However, it was shown that torque limits of the joint motors restrict the feasible solutions for the arm configuration [23]. It is therefore advantageous to exploit passive stiffness for assistance, as Hogan originally asserted [13].

The paper is organised as follows:

- First we review the calculation of the Jacobian matrix both algebraically and with respect to arm geometry,
- Second, we propose an objective function for optimising stiffness and compute its maxima and minima with respect to properties of a serial link arm.
- Then we apply these results for optimal stiffness and compliance to observations of humans in neurophysiology, and demonstrate their application in robotics.
- Finally, we discuss some implications and directions for future research given the results in this paper.

## Arm geometry & the Jacobian matrix

The derivative of the forward kinematics Eq 2 is obtained from the chain rule. Using this approach the Jacobian is regarded as a matrix of partial derivatives with respect to each joint:

$$\begin{array}{c} \text{J}\left(\text{q}\right)=\frac{\partial \text{g}}{\partial \text{q}}=[\begin{array}{ccc} \frac{\partial {\text{g}}_{1}}{\partial {\text{q}}_{1}} & \cdots & \frac{\partial {\text{g}}_{1}}{\partial {\text{q}}_{n}}\\ \vdots & \ddots & \vdots \\ \frac{\partial {\text{g}}_{m}}{\partial {\text{q}}_{1}} & \cdots & \frac{\partial {\text{g}}_{m}}{\partial {\text{q}}_{n}} \end{array}]\in R^{\text{m}\times \text{n}}. \end{array}$$ (8)

In practice, particularly for robotic control, the Jacobian may be computed numerically from known geometric properties of the forward kinematics [32]. Joints are classified in 2 fundamental types:

1. Revolute joints, which rotate about a given axis of actuation $\hat{\text{a}}\in R^{\text{m}}$, and
2. Prismatic joints, which translate along an axis $\hat{\text{a}}$.

**Fig 1:**
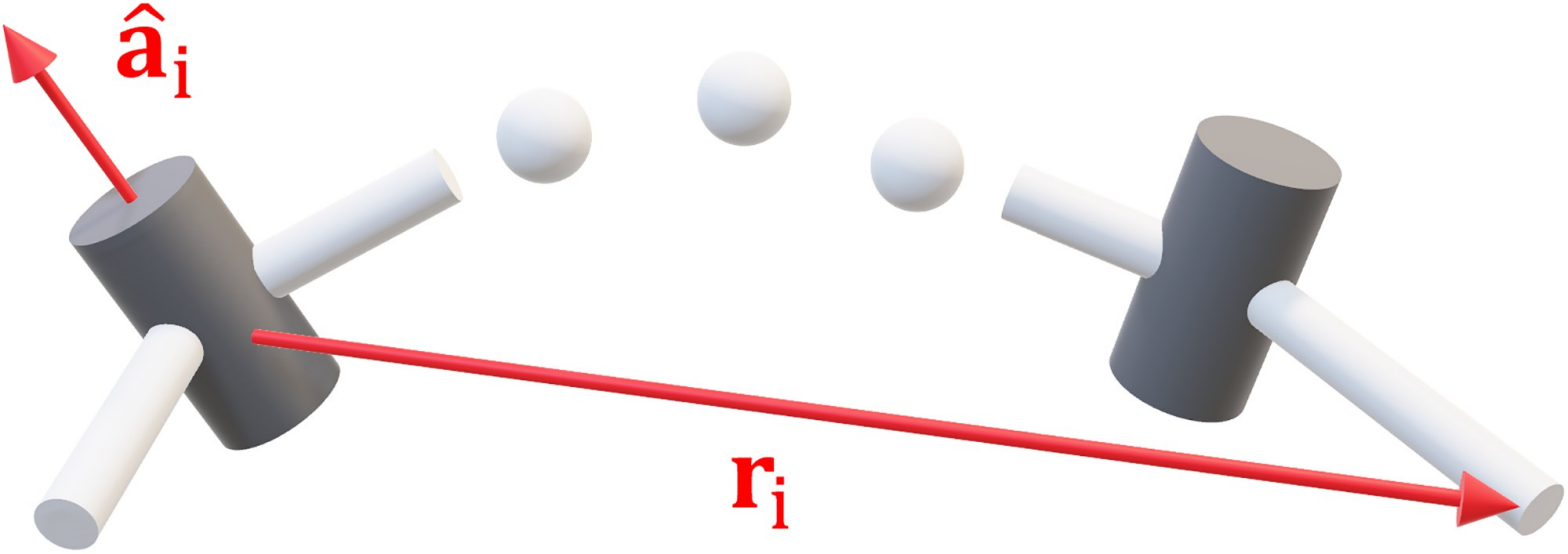
Geometry of the Jacobian matrix. The i^th^ column of the Jacobian is formed using the joint axis ${\hat{\text{a}}}_{\text{i}}$ and translation vector to the endpoint **r**_i_.

More complex joints are formed through different combinations of these two (for example, a ball joint may be modelled as 3 revolute joints). For m = 6 the i^th^ column of the Jacobian can be written in terms of the joint axis $\hat{\text{a}}\in R^{3}$ and the translation vector $\text{r}\in R^{3}$ from the joint to the endpoint (Fig 1):

$$\begin{array}{c} {\text{J}}_{i}=\{\begin{array}{cc} \left[\begin{array}{c} {\hat{a}}_{i}\times r_{i}\\ {\hat{a}}_{i} \end{array}\right] & \text{if}\;\text{i}\;\text{is}\;\text{a}\;\text{revolute}\;\text{joint}\\ \;[\begin{array}{c} {\hat{a}}_{i}\\ 0 \end{array}] & \text{if}\;\text{i}\;\text{is}\;\text{a}\;\text{prismatic}\;\text{joint} \end{array} \end{array}$$ (9)

Likewise, we can also express the derivative of the Jacobian using the same vectors [33, 34]:

$$\begin{array}{c} \frac{\partial {\text{J}}_{\text{i}}}{\partial {\text{q}}_{\text{j}}}=\{\begin{array}{cc} \left[\begin{array}{c} {\hat{\text{a}}}_{\text{j}}\times ({\hat{\text{a}}}_{\text{i}}\times {\text{r}}_{\text{i}})\\ {\hat{\text{a}}}_{\text{j}}\times {\hat{\text{a}}}_{\text{i}} \end{array}\right] & \begin{array}{c} \text{if}\;\text{i}\;\text{is}\;\text{revolute,}\\ \text{j}\;\text{is}\;\text{revolute}\;\&\text{j}\leq \text{i} \end{array}\\ \left[\begin{array}{c} {\hat{\text{a}}}_{\text{j}}\times ({\hat{\text{a}}}_{\text{i}}\times {\text{r}}_{\text{i}})\\ 0 \end{array}\right] & \begin{array}{c} \text{if}\;\text{i}\;\text{is}\;\text{revolute,}\\ \text{j}\;\text{is}\;\text{revolute}\;\text{or}\;\text{prismatic}\;\&\text{j}>\text{i} \end{array}\\ \;[\begin{array}{c} {\hat{\text{a}}}_{\text{j}}\times {\hat{\text{a}}}_{\text{i}}\\ 0 \end{array}] & \begin{array}{c} \text{if}\;\text{i}\;\text{is}\;\text{revolute,}\\ \text{j}\;\text{is}\;\text{prismatic}\;\&\text{j}\leq \text{i} \end{array}\\ \;[\begin{array}{c} 0\\ 0 \end{array}] & \text{otherwise.} \end{array} \end{array}$$ (10)

Note that the partial derivative of the Jacobian is not symmetric:

$$\begin{array}{c} \frac{\partial {\text{J}}_{\text{i}}}{\partial {\text{q}}_{\text{j}}}\neq \frac{\partial {\text{J}}_{\text{j}}}{\partial {\text{q}}_{\text{i}}}. \end{array}$$ (11)

Given Eq 9 we could then express Eq 3 as a sum of column vectors weighted by the joint displacements $\delta {\text{q}}_{\text{i}}\in R$:

$$\begin{array}{c} \delta \text{x}={\text{J}}_{1}\delta {\text{q}}_{1}+\cdots +{\text{J}}_{\text{n}}\delta {\text{q}}_{\text{n}}. \end{array}$$ (12)

By analysing the column vectors of the Jacobian, we can understand the contribution of individual joints to the total endpoint displacement and hence its stiffness.

## Optimal configurations for stiffness & compliance

### Conditions for optimality

We require a scalar quantity with which to optimise the stiffness. First we note that the joint torques from a given deflection are obtained by substituting Eqs 4b in to 1: ***τ*** = **J**^T^**K**_c_Δ**x**. Then by projecting this vector on to itself (i.e. the dot product) we obtain the sum-of-squares as a scalar:

$$\begin{array}{c} \text{p}\left(\text{q}\right)=\frac{1}{2}\tau^{\text{T}}\tau \end{array}$$ (13a)

$$=\frac{1}{2}{\text{w}}^{\text{T}}{\text{JJ}}^{\text{T}}\text{w}$$ (13b)

$$=\frac{1}{2}\delta {\text{xK}}_{\text{c}}{\text{JJ}}^{\text{T}}{\text{K}}_{\text{c}}\delta \text{x}.$$ (13c)

We halve this quantity to make its derivatives neater. We can interpret Eq 13 as the magnitude of the joint torques for a given deflection of the endpoint. This is half the stiffness ellipsoid, and its longest principal radius gives the direction in which the arm has the *least* deflection when under load. We can see that the stiffness ellipsoid takes the force ellipsoid **JJ**^T^ (something configuration dependent) and compounds the Cartesian stiffness matrix **K**_c_ (something designed). It combines the inherent mechanical properties of the arm with the restoring forces that oppose displacement at the endpoint.

We can take the derivative of Eq 13 and substitute in Eq 6 to obtain:

$$\begin{array}{c} \frac{\partial \text{p}}{\partial \text{q}}=(\frac{\partial \tau }{\partial \text{q}}{)}^{\text{T}}\tau =({\text{K}}_{\text{a}}+{\text{K}}_{\text{p}}){\text{J}}^{\text{T}}\text{w}. \end{array}$$ (14)

A given joint position **q** is an extremum of Eq 13 if ∂p/∂**q** = **0**. This constitutes the first order optimality condition. Taking the derivative a second time produces the Hessian matrix:

$$\begin{array}{c} \text{H}\left(\text{q}\right)=\frac{\partial^{2}\text{p}}{\partial {\text{q}}^{2}}=(\frac{\partial^{2}\tau }{\partial {\text{q}}^{2}}{)}^{\text{T}}\tau +(\frac{\partial \tau }{\partial \text{q}}{)}^{\text{T}}(\frac{\partial \tau }{\partial \text{q}}). \end{array}$$ (15)

An extremum of Eq 13 is a local minimum if Eq 15 is positive definite: **H(q)** ≻ 0. Conversely, it is a local maximum if Eq 15 is negative definite: **H(q)** ≺ 0. These constitute the second order optimality conditions. We want to find for what configurations of the arm that Eq 13 is maximized or minimized. We know that the Jacobian and its derivative can be expressed in terms of the joint axis and translation vectors Eqs 9 and 10. We wish to find what properties of the axis vector $\hat{\text{a}}$ and displacement vector **r** produce stiffness or compliance at the endpoint.

### Optimal stiffness

Since Eq 13 equates to a sum-of-squares, it is always greater than or equal to zero p(**q**) ≥ 0 ∀**q**. Thus, from inspection, we can see that it is a minimum when:

$$\begin{array}{c} {\text{J}}^{\text{T}}\text{w}=0. \end{array}$$ (16)

Likewise, the gradient Eq 14 is also zero and hence this constitutes an extrema of Eq 13. For this case the Hessian Eq 15 reduces to:

$$\begin{array}{c} \text{H}\left(\text{q}\right)=(\frac{\partial \tau }{\partial \text{q}}{)}^{\text{T}}(\frac{\partial \tau }{\partial \text{q}}) \end{array}$$ (17)

which is positive definite. This confirms that **J**^T^**w** = **0** is a minimum of Eq 13.

The fact that **J**^T^**w** = **0** for **w** ≠ **0** implies that Jacobian **J** and hence the stiffness ellipsoid **K**_c_**JJ**^T^**K**_c_ are singular. We can apply the singular value decomposition (SVD) to provide further insight in to its consequences of a singularity:

$$\begin{array}{c} {\text{K}}_{\text{c}}{\text{JJ}}^{\text{T}}{\text{K}}_{\text{c}}={\text{USV}}^{\text{T}} \end{array}$$ (18)

where **U**, V∈O(6) are orthogonal matrices. The singular values equate to the squared inverse of the principle radii of the ellipsoid:

$$\text{S}=[\begin{array}{ccc} {\text{s}}_{\text{max}} & & \\ & \ddots & \\ & & {\text{s}}_{\text{min}} \end{array}]=[\begin{array}{ccc} {\text{r}}_{\text{min}}^{-\text{2}} & & \\ & \ddots & \\ & & {\text{r}}_{\text{max}}^{-\text{2}} \end{array}]$$ (19)

They are always arranged from largest to smallest. Hence, the smallest singular value is proportional to the longest axis of the Cartesian stiffness ellipsoid. As the arm approaches a singular configuration, the length of the longest axis will grow to infinity:

$$\underset{{\text{s}}_{\text{min}}\to \text{0}}{\text{lim}}{\text{r}}_{\text{max}}=\infty .$$

**Fig 2:**
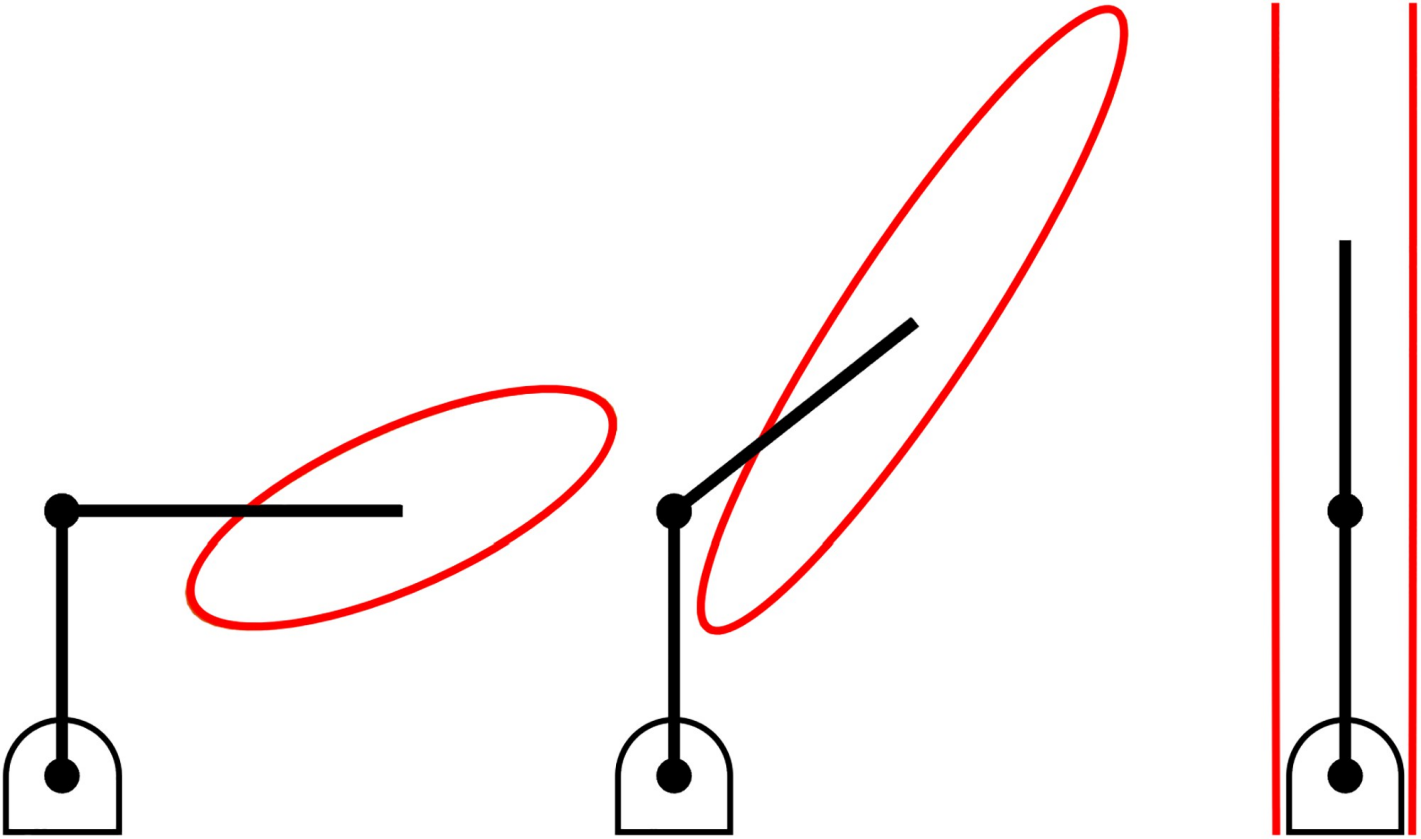
The Cartesian stiffness ellipsoid of a serial link mechanism. As the mechanism approaches a singular configuration the longest axis of the Cartesian stiffness ellipsoid grows to infinity.

The arm will have theoretically infinite stiffness in this direction when singular, as illustrated in Fig 2 (assuming the individual rigid bodies that comprise the arm themselves have infinite tensile or compressive strength).

It had been hypothesized in neurophysiology that one of the goals in human manipulation tasks is to minimize energy consumption: “the CNS [central nervous system] attempts to both maintain a minimum level of stability and minimize energy expenditure” [2]. This seems to imply a zero-sum between the two objectives. While it is true that muscle contraction stiffens the arm, it is also metabolically costly [35]. When singular, the arm requires zero joint torque (i.e. muscle contraction) to withstand external forces since ***τ*** = **J**^T^**w** = **0**. Maximising stiffness and minimising energy expenditure can be achieved simultaneously. They are the same strategy.

Suppose that we apply a 3D wrench of forces and moments to the endpoint:

$$\text{w}=[\begin{array}{c} \text{f}\\ \text{m} \end{array}]$$

where $\text{f}\in R^{3}$ are the linear forces (N), and $\text{m}\in R^{3}$ are the moments (Nm). From Eq 16 it must hold that the projection of all column vectors of the Jacobian and the wrench must be zero:

$$\begin{array}{c} {\text{J}}_{\text{i}}^{\text{T}}\text{w}=0\;\forall \text{i}. \end{array}$$ (20)

In the next two sections this result is examined with respect to revolute and prismatic joints.

#### For revolute joints

If we take the definition of the column vector of a revolute joint in Eq 9 and substitute it in to Eq 20 the result is:

$$\begin{array}{c} ({\hat{\text{a}}}_{\text{i}}\times {\text{r}}_{\text{i}}{)}^{\text{T}}\text{f}+{\hat{\text{a}}}_{\text{i}}^{\text{T}}m=0. \end{array}$$ (21)

From inspection, one possible condition for this identity is that each term is zero: $({\hat{\text{a}}}_{\text{i}}\times {\text{r}}_{\text{i}}{)}^{\text{T}}\text{f}={\hat{\text{a}}}_{\text{i}}^{\text{T}}m=0$. The dot product, or projection of the vectors, is zero and hence they must be orthogonal:

$${\hat{\text{a}}}_{\text{i}}+{\text{r}}_{\text{i}}⊥\text{f}$$ (22a)

$${\hat{\text{a}}}_{\text{i}}⊥\text{m}.$$ (22b)

**Fig 3:**
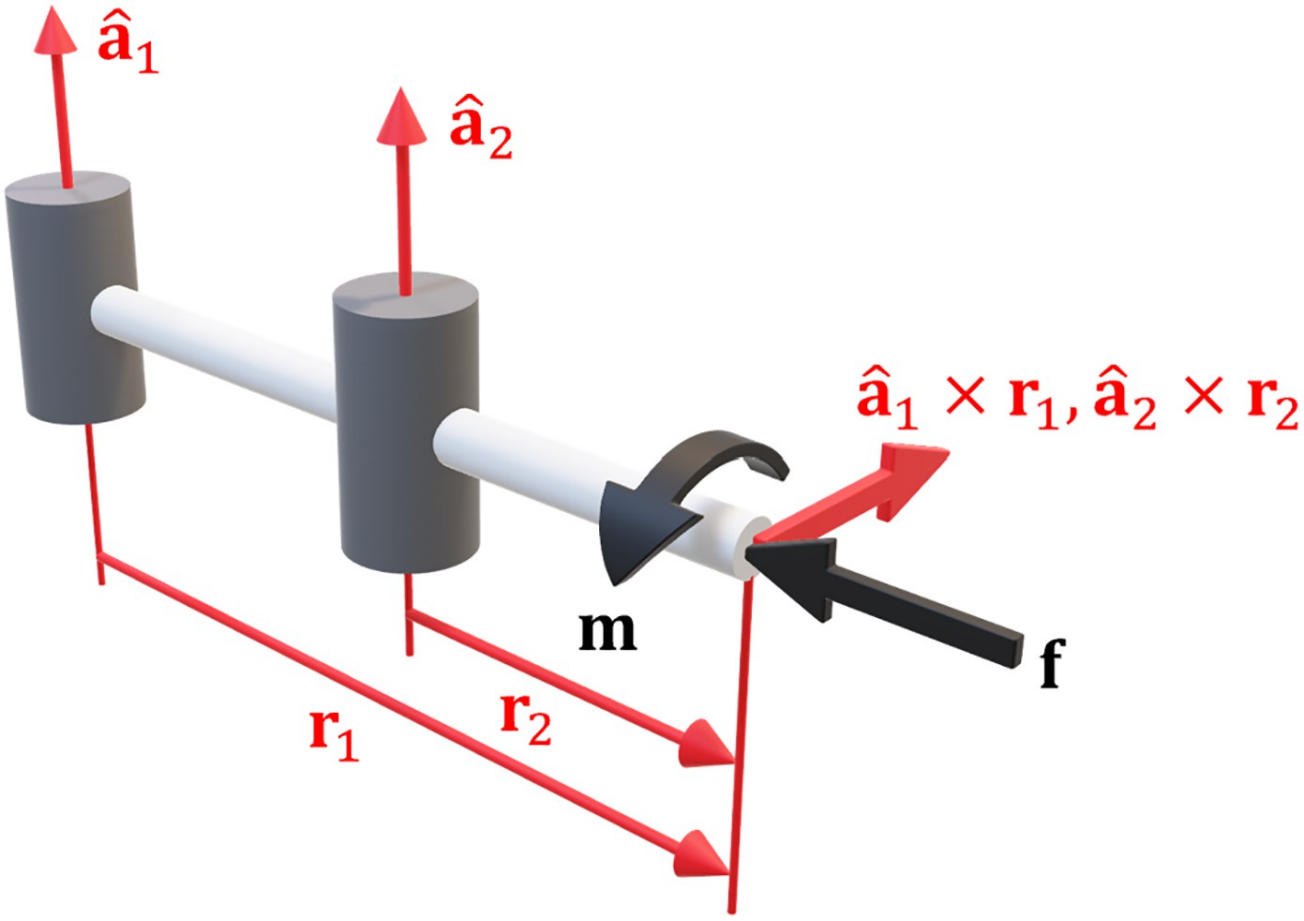
Optimal stiffness for revolute joints. In this configuration the forces and moments on the endpoint produce zero joint torque.

Any moment **m** applied to the endpoint must be orthogonal to the joint axis ${\hat{\text{a}}}_{\text{i}}$ and hence it produces zero joint torque. The moment produced by the linear forces is **r**_i_ × **f**, so Eq 22a can be rearranged to say that ${\text{r}}_{\text{i}}\times \text{f}⊥{\hat{\text{a}}}_{\text{i}}$. It produces zero joint torque and hence zero deflection ensues. An example of an optimally stiff configuration is illustrated in Fig 3.

Equation Eq 21 can also be rearranged as:

$$({\text{r}}_{\text{i}}\times \text{f}{)}^{\text{T}}{\hat{\text{a}}}_{\text{i}}+{\text{m}}^{\text{T}}{\hat{\text{a}}}_{\text{i}}=0$$ (23a)

$$({\text{r}}_{\text{i}}\times \text{f}+\text{m}{)}^{\text{T}}{\hat{\text{a}}}_{\text{i}}=0.$$ (23b)

Thus another possible condition is:

$$\text{m}=-{\text{r}}_{\text{i}}\times \text{f}\text{.}$$ (24)

**Fig 4:**
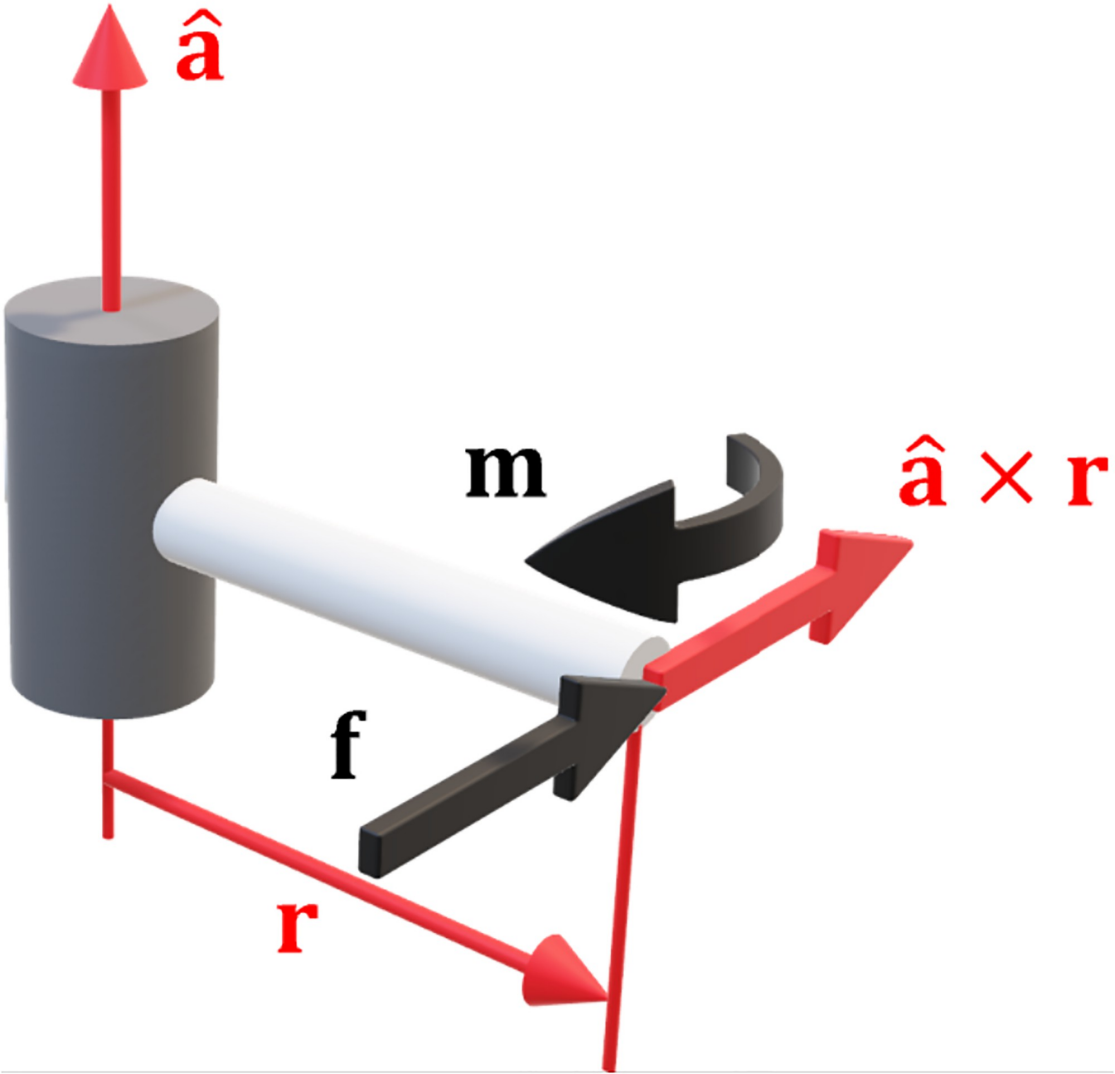
Alternate optimal stiffness for a revolute joint. In this configuration the endpoint forces and moments negate each other.

The moments produced by the force are equal and opposite to the moment on the endpoint. The mechanism has optimal *configuration* dependent stiffness since there is zero net joint torque and hence zero deflection. An example of this is illustrated in Fig 4.

#### For prismatic joints

Using the column vector of the Jacobian for a prismatic joint Eq 9, and substituting in to Eq 20 leads to:

$$\begin{array}{c} {\hat{\text{a}}}_{\text{i}}^{\text{T}}\text{f}=0. \end{array}$$ (25)

The projection of the forces on to the joint axis is zero, so the vectors must be orthogonal:

$$\begin{array}{c} {\hat{\text{a}}}_{\text{i}}⊥\text{f}. \end{array}$$ (26)

**Fig 5:**
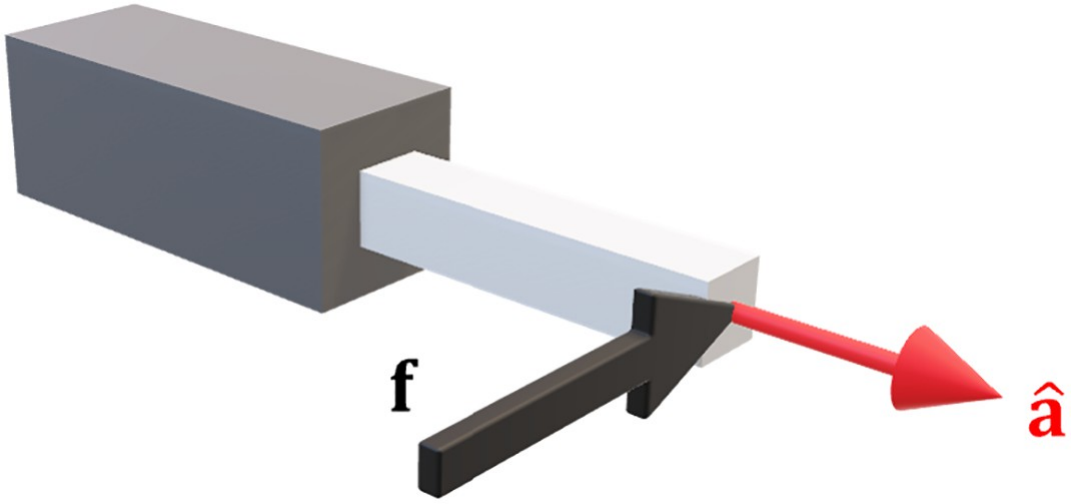
Optimal stiffness for a prismatic joint. Applied forces must be orthogonal to the axis of actuation.

This result is illustrated in Fig 5.

### Optimal compliance

From inspection of Eq 14 another possible condition for an extrema of Eq 13 is that:

$$\begin{array}{c} {\text{K}}_{\text{a}}+{\text{K}}_{\text{p}}=0. \end{array}$$ (27)

A potential solution here is that **K**_a_ = −**K**_p_. However, it is proven in the S1 Appendix that this equality is impossible. Therefore, the only other possibility is that:

$$\begin{array}{c} {\text{K}}_{\text{a}}={\text{K}}_{\text{p}}=0. \end{array}$$ (28)

It is difficult to prove that this is a maxima of Eq 13 since the derivation of the Hessian Eq 15 becomes too complex. However, we can intuit that this constitutes minimum stiffness / maximum compliance. The fact that **K**_a_ = **0** means that **K**_c_ = **0** and the arm does not actively apply any restoring forces when the endpoint is displaced. It complies with any forces applied to it. Then, for **K**_p_ = **0**, it must hold that:

$$\begin{array}{c} {\text{K}}_{\text{p}}=\frac{\partial {\text{J}}^{\text{T}}}{\partial \text{q}}\text{w}=0 \end{array}$$ (29)

and hence:

$$\begin{array}{c} \frac{\partial {{\text{J}}_{\text{i}}}^{\text{T}}}{\partial {\text{q}}_{\text{j}}}\text{w}=0\;\forall \;\text{i},\text{j}. \end{array}$$ (30)

We can use the derivatives of the Jacobian Eq 10 to further analyse this result.

#### For revolute joints

Substituting the revolute case of Eq 10 in to Eq 30 yields:

$$\begin{array}{c} ({\hat{\text{a}}}_{\text{j}}\times ({\hat{\text{a}}}_{\text{i}}\times {\text{r}}_{\text{i}}){)}^{\text{T}}\text{f}+({\hat{\text{a}}}_{\text{j}}\times {\hat{\text{a}}}_{\text{i}}{)}^{\text{T}}\text{m}=0 \end{array}$$ (31a)

$$\begin{array}{c} (({\hat{\text{a}}}_{\text{i}}\times {\text{r}}_{\text{i}})\times \text{f}{)}^{\text{T}}{\text{a}}_{\text{j}}+({\hat{\text{a}}}_{\text{i}}\times \text{m}{)}^{\text{T}}{\text{a}}_{\text{j}}=0 \end{array}$$ (31b)

One potential solution here is that:

$$\begin{array}{c} ({\hat{\text{a}}}_{\text{i}}\times {\text{r}}_{\text{i}})\times \text{f}=\hat{\text{a}}\times \text{m}=0. \end{array}$$ (32)

The cross product of the vectors must be zero, meaning they are parallel:

$$\begin{array}{c} {\hat{\text{a}}}_{\text{i}}\times {\text{r}}_{\text{i}}\|\text{f} \end{array}$$ (33a)

$$\begin{array}{c} {\hat{\text{a}}}_{\text{i}}\|\text{m}. \end{array}$$ (33b)

**Fig 6:**
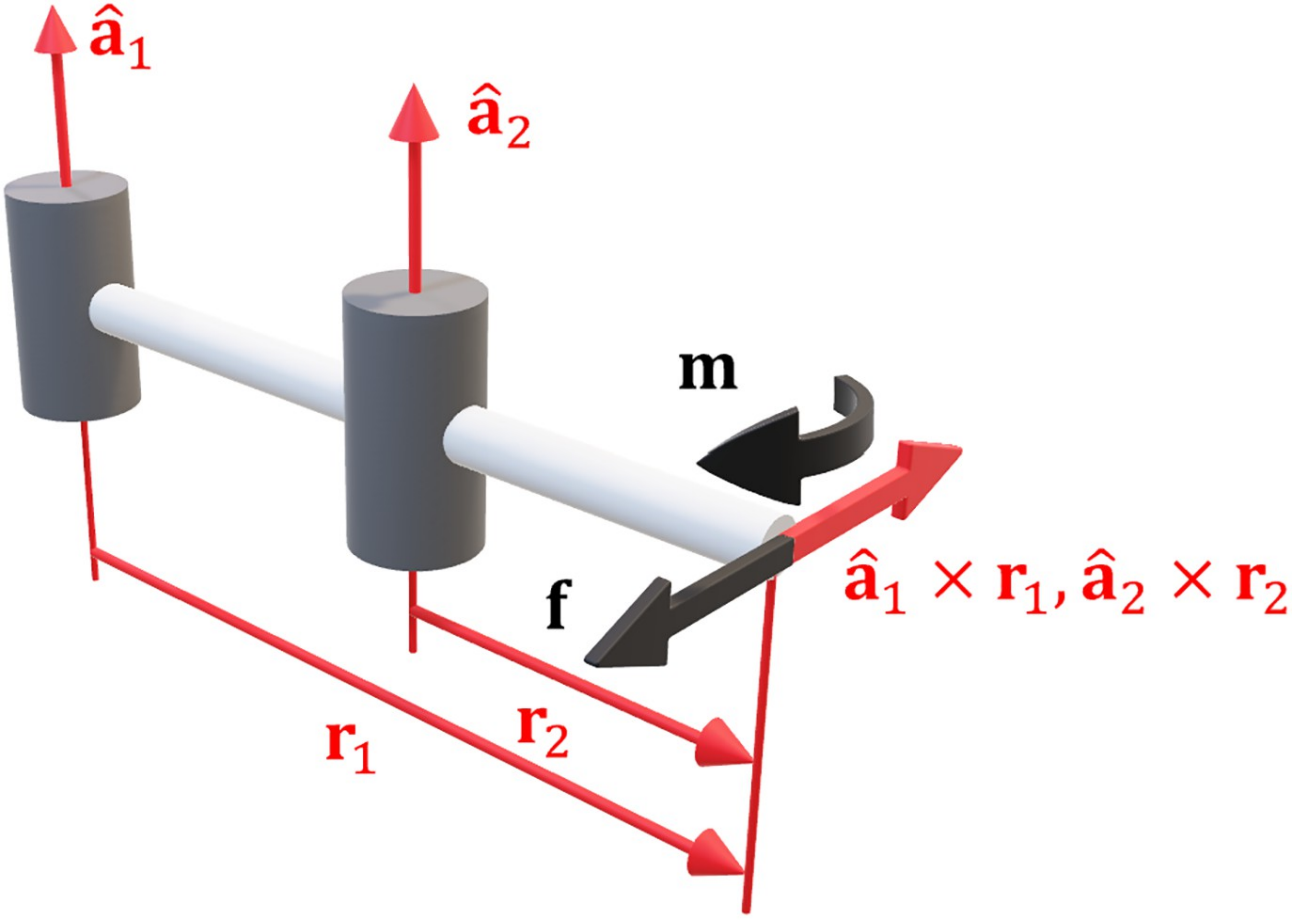
Optimal compliance in revolute joints. The moments from the endpoint forces are parallel to the joint axes, maximising joint torque and hence joint deflection.

The moments on the endpoint **m** are parallel to the joint axis ${\hat{\text{a}}}_{\text{i}}$ and hence project the maximum torque in to the joint. Similarly, the moment produced by the forces **r**_i_ × **f** must also be parallel to ${\hat{\text{a}}}_{\text{i}}$. Maximising the induced joint torque maximises the possible deflection thus producing maximum compliance. An example of an arm in an optimally compliant configuration is illustrated in Fig 6.

By rearranging Eq 31 further we can get:

$$\begin{array}{c} ((({\text{r}}_{\text{i}}\times \text{f})-\text{m})\times {\hat{\text{a}}}_{\text{i}}{)}^{\text{T}}{\hat{\text{a}}}_{\text{j}} \end{array}$$ (34)

such that another possible condition is:

$$\begin{array}{c} {\text{r}}_{\text{i}}\times \text{f}-\text{m}=0. \end{array}$$ (35)

The moments resulting from the linear forces **r**_i_ × **f** are in the same direction as the pure moments at the endpoint:

$$\begin{array}{c} \text{m}={\text{r}}_{\text{i}}\times \text{f}. \end{array}$$ (36)

If Eq 33 holds then the combination of forces and moments amplifies the resulting joint torque. This will produce maximum deflection and thus maximum compliance. This is the opposite of the scenario for stiffness Eq 24, which conforms with intuition.

#### For prismatic joints

If we substitute the prismatic case for Eq 10 in to Eq 30 to one obtains:

$$\begin{array}{c} ({\hat{\text{a}}}_{\text{j}}\times {\hat{\text{a}}}_{\text{i}}{)}^{\text{T}}\text{f}=0 \end{array}$$ (37a)

$$({\hat{\text{a}}}_{\text{i}}\times \text{f}{)}^{\text{T}}{\hat{\text{a}}}_{\text{j}}=0.$$ (37b)

For this equation to be satisfied then the linear forces must be parallel to the axis of the joint:

$${\hat{\text{a}}}_{\text{i}}\|\text{f}.$$ (38)

**Fig 7:**
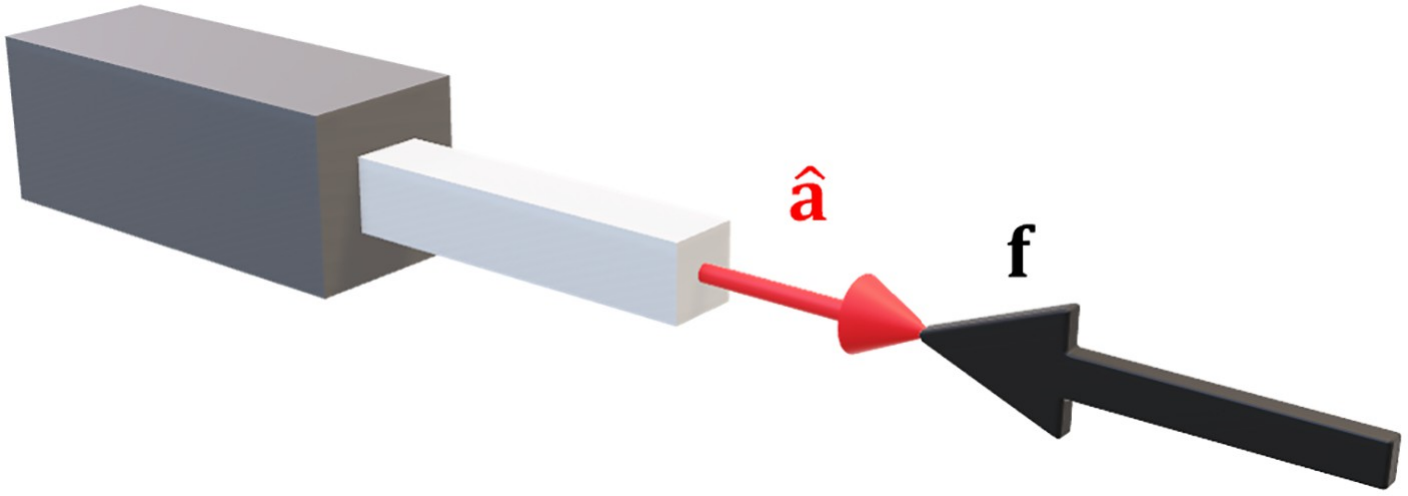
Optimal compliance in a prismatic joint. The applied forces are orthogonal to the axis of actuation.

As we would expect, the case for maximum compliance is opposite that for maximum stiffness Eq 26. An illustration of maximum compliance for a prismatic joint is given in Fig 7.

## Case studies

### Explaining observations in humans

Numerous observations have been made in neurophysiology about the shape of the arm in relationship to the Cartesian stiffness ellipsoid. Stiffness is estimated through experiment by using a mechanical device to generate forces on the hand from which the ensuing deflection is measured. However, pioneering research in engineering is able to estimate stiffness from the observed arm configuration [29, 30]. In the same manner, we can use the results for optimal stiffness Eq 22 and compliance Eq 33 to infer stiffness, and retroactively explain neurophysiology.

For example, [11] wrote:

“… the stiffness is… more anisotropic (elongated) in distal [outstretched] positions; the direction of the maximum stiffness is approximately oriented along a radial line joining the hand to the shoulder.”

**Fig 8:**
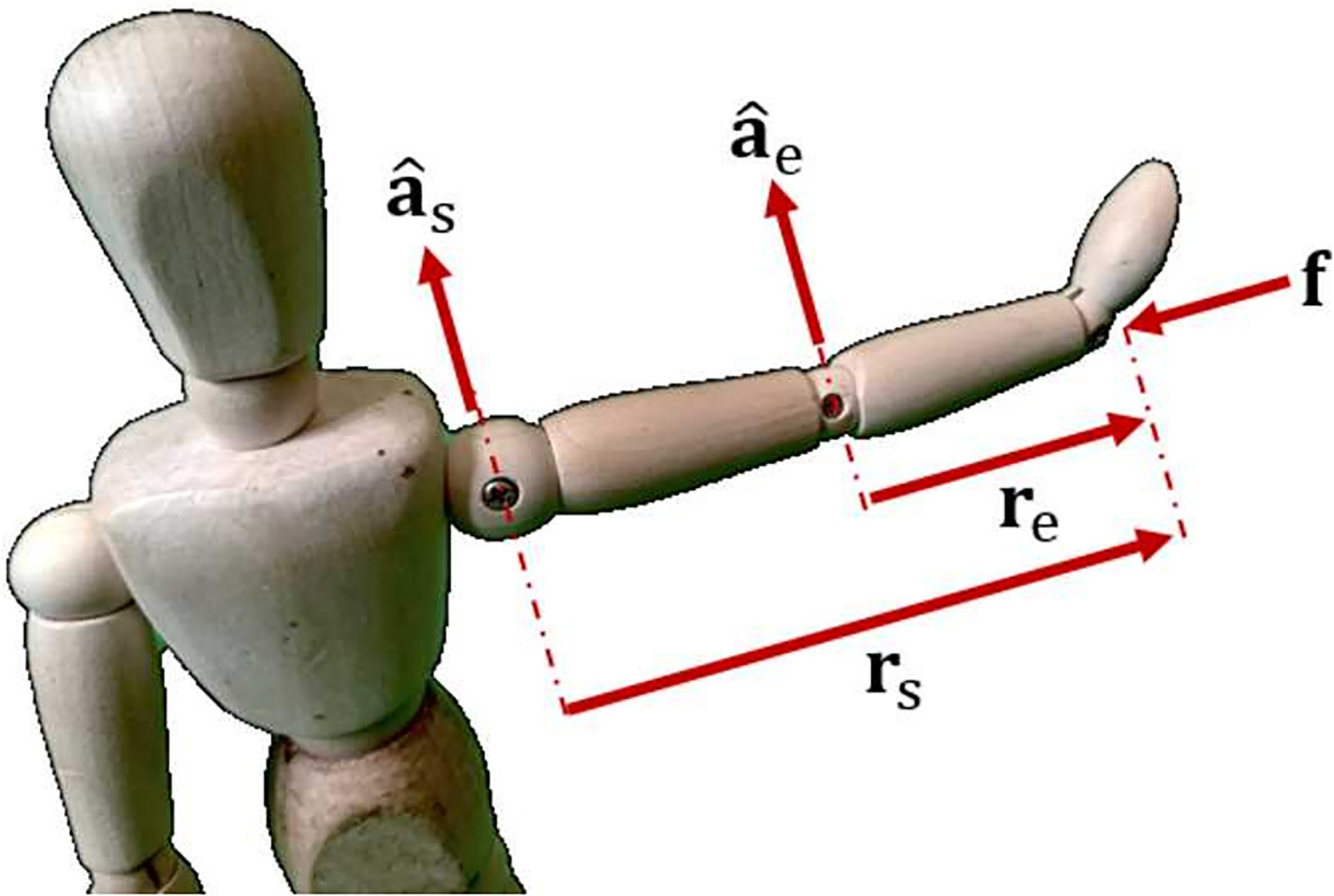
A singular configuration in the human arm. An outstretched arm leads to a kinematic singularity in the direction from the shoulder to the hand. This results in maximum stiffness.

Fig 8 shows an arm in an outstretched position where:

- **r**_s_ is the radial line from the shoulder to the hand,
- **r**_e_ is the radial line from the elbow to the hand,
-
${\hat{\text{a}}}_{\text{s}}$ and ${\hat{\text{a}}}_{\text{e}}$ are the axes of the shoulder and elbow, respectively, perpendicular to the horizontal plane, and
- **f** is a force applied parallel to the horizontal plane.

In this situation the vectors **r**_s_ and **r**_e_ are parallel. The arm is in a singular configuration, which is one of the results for maximum stiffness. The force vector in Fig 8 is also orthogonal to the vectors ${\hat{\text{a}}}_{\text{s}}\times {\text{r}}_{\text{s}}$ and ${\hat{\text{a}}}_{\text{e}}\times {\text{r}}_{\text{e}}$ which adheres to the optimality conditions previously derived Eq 22.

Milner would describe a similar scenario regarding the stiffness ellipsoid relative to an outstretched arm [4]:

“… [the] subjects’ ability to maintain a precise position of the hand progressively deteriorated except in the K_4_ condition. Because the elbow was extended at this hand position, endpoint stiffness was relatively high in the anterior-posterior direction…”

**Fig 9:**
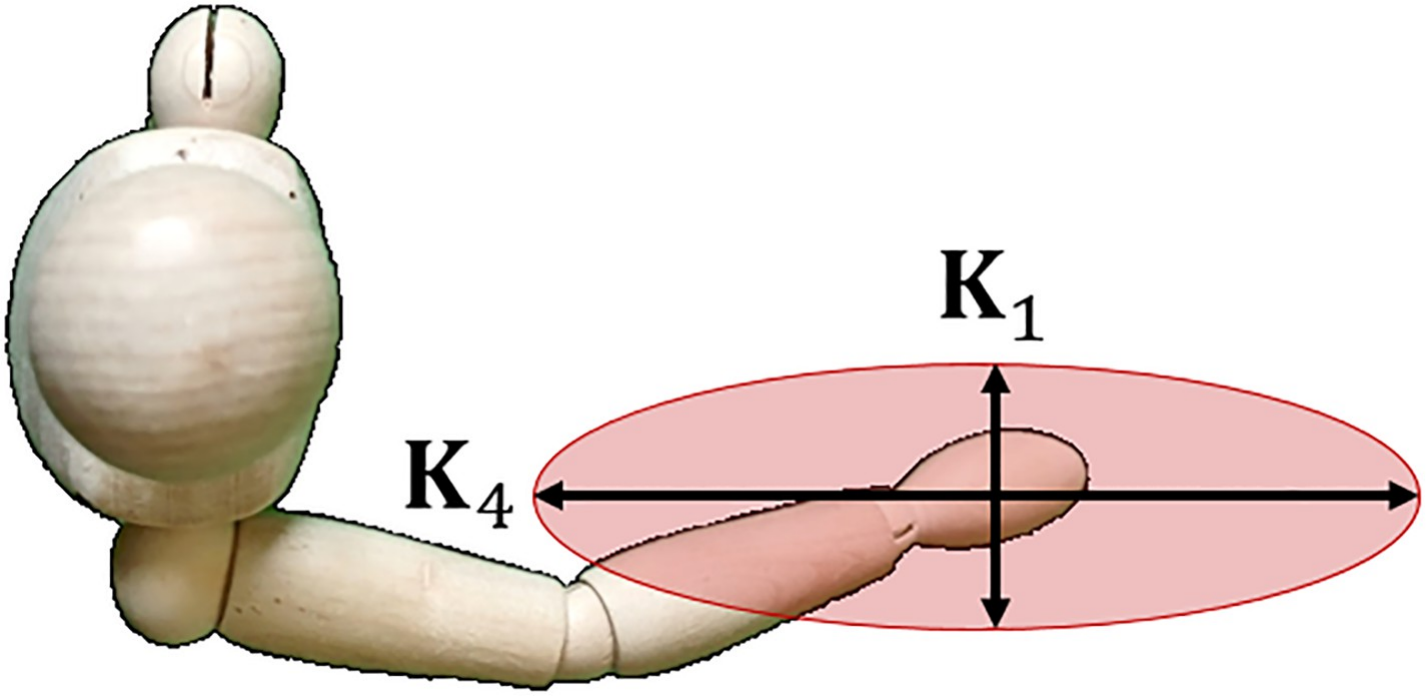
Stiffness ellipsoid in a human arm. The principle radii correspond to directions of maximum stiffness, and maximum compliance respectively.

Fig 9 shows an illustration of this description. It is the same situation as previously described. When the elbow is extended, the arm forms a straight line and is in a singular configuration. Forces applied in the K_4_ direction thus produce minimal joint torque resulting in increased stiffness as previously described. From the same article Milner also wrote that:

“…endpoint stiffness was greatly diminished in the orthogonal direction K_1_ and this corresponded to the greatest drop in success score as force field strength increased.”

This description matches the conditions for optimal compliance Eq 33. The arm has minimal passive stiffness in the K_1_ direction, and is limited to capacity of the muscles in generating joint torque. If the force is too high and/or prolonged, the muscles will eventually succumb to fatigue.

**Fig 10:**
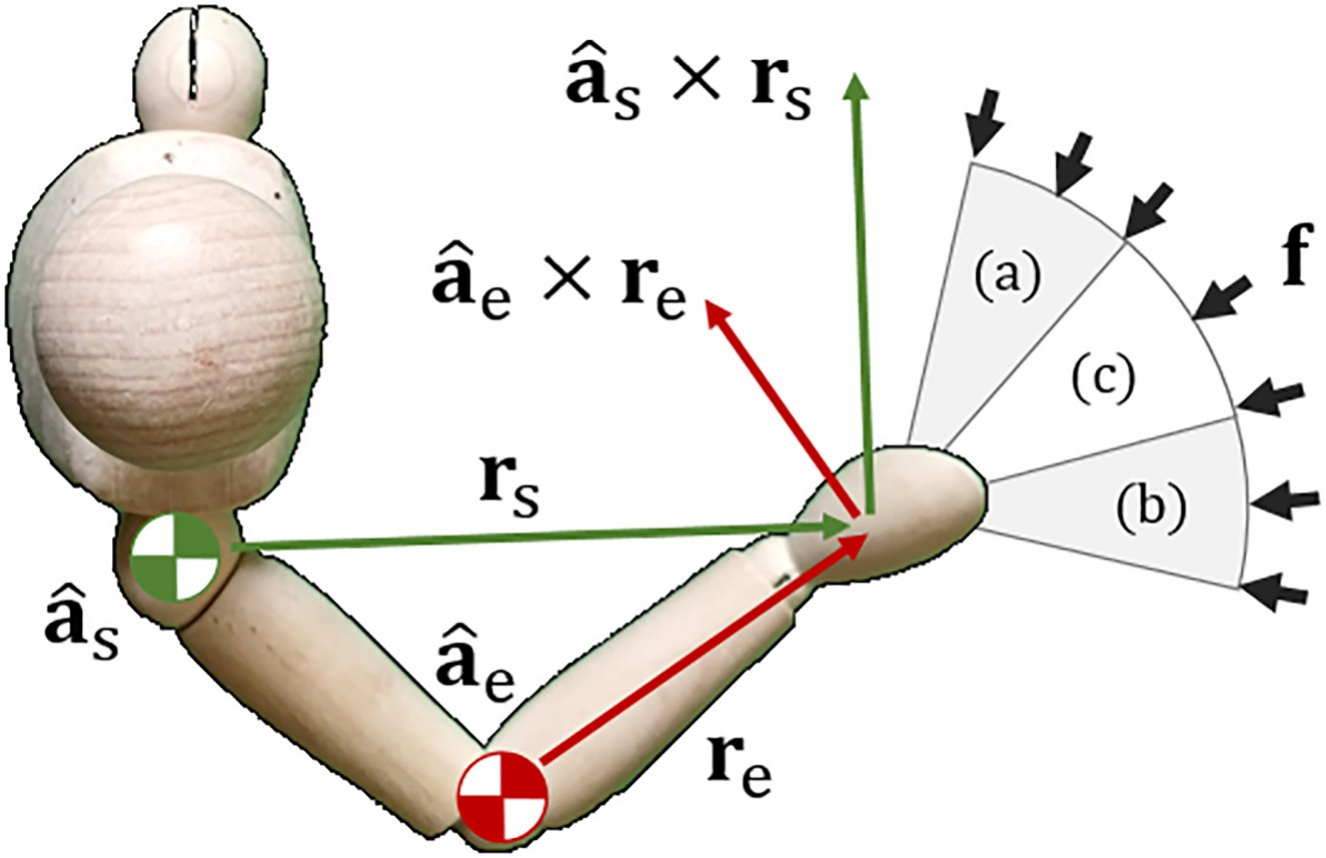
Stiffness is relative to the direction of applied forces. Forces in region (a) lead to shoulder muscle activation, forces in region (b) lead to elbow muscle activation, and forces in region (c) create both.

Fig 10 shows an illustration of a human with a bend in the elbow. Experiments were done in which forces were applied in different directions resulting in different muscle activation [10]:

(a) predominantly shoulder muscles,
(b) predominantly elbow muscles, and
(c) a combination of the two.

Forces in region (a) are roughly orthogonal to the line of motion that the elbow produces as the hand $\text{f}⊥{\hat{\text{a}}}_{\text{e}}\times {\text{r}}_{\text{e}}$. This results in minimal elbow torque which requires the shoulder to do most of the work. Conversely, in region (b), the forces are orthogonal to the shoulder joint actuation $\text{f}⊥{\hat{\text{a}}}_{\text{s}}\times {\text{r}}_{\text{s}}$. This necessitates that the elbow bear most of the forces. No orthogonality is present in region (c) and hence a combination of elbow and shoulder joint torque is required. These observations are consistent with the optimality conditions previously derived Eqs 22 and 33.

### Planning in robotic tasks

In this section we show how the performance criterion proposed prior can be used for task planning. There are many scenarios where exploiting the passive stiffness in a robotic arm can be used to improve task performance. For example, it was shown that a simple change in configuration could improve the surface finish in a polishing task by absorbing vibrations [17]. More recently, an admittance control framework was used for machining bone for use in hip replacement surgery [36]. Adopting an appropriate arm posture could further enhance the admittance control.

Whilst the forward kinematics gives the pose of the endpoint as a function of joint angles **x = g(q)** Eq 2, inverse kinematics involves finding the joint angles that satisfy a given endpoint pose: **q** = **g**^−1^(**x**). This can be achieved through closed form solutions or mathematical optimisation. The forward kinematics mapping is always unique, whereas the inverse kinematics may have several solutions. For example, the UR3, UR5, and UR10 robotic arms from Universal Robot have 8 solutions for the inverse kinematics [37]. Given these solutions for the inverse kinematics, we can use Eq 13 to evaluate the relative stiffness for each in the direction $\hat{\text{x}}$. The configuration that gives the best result (be it stiffness or compliance) can then be chosen for use (for example, a compliant posture in addition to the active feedback control in [20]).

**Fig 11:**
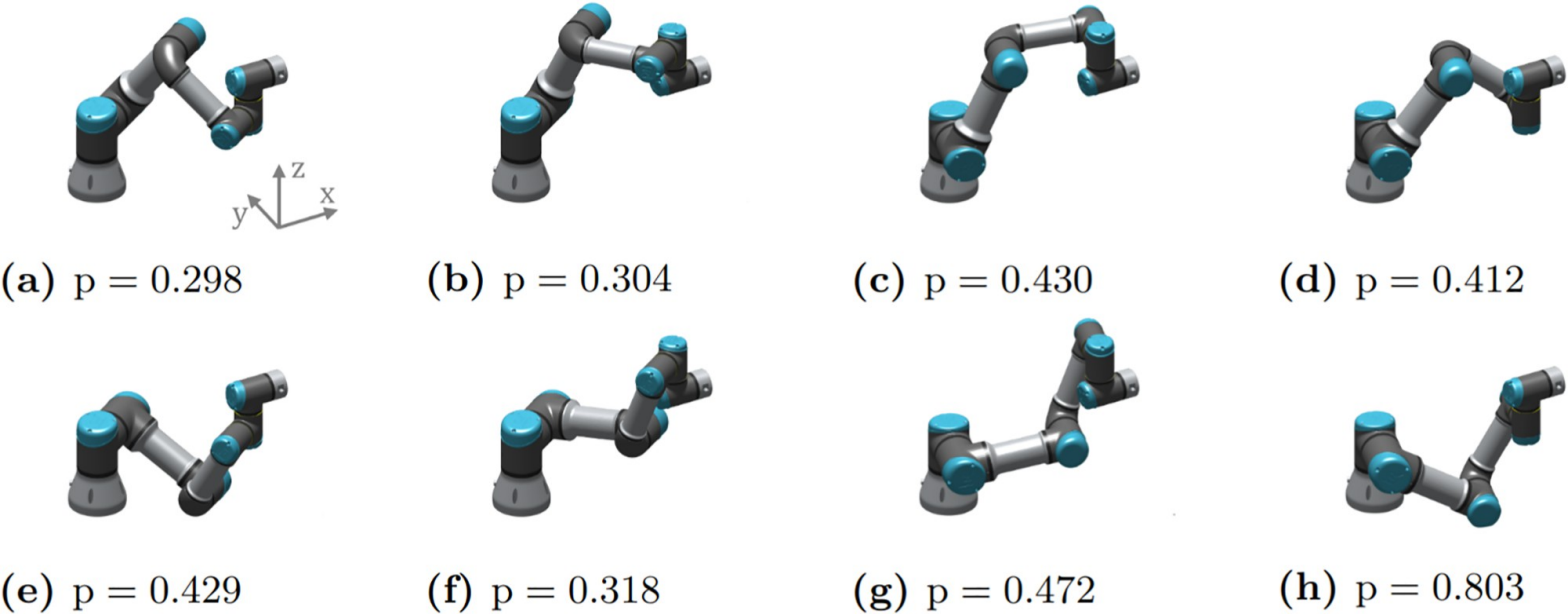
The 8 inverse kinematics solutions for the UR3 robot arm. The relative stiffness in the x-direction is compute for each configuration using Eq 13.

Fig 11 shows the 8 inverse kinematics solutions of the UR3 robot for a given endpoint pose. Eq 13 was computed for each in the direction $\hat{\text{x}}=[\begin{array}{llllll} 1 & 0 & 0 & 0 & 0 & 0 \end{array}{]}^{\text{T}}$, i.e. how stiff the robot is in the x-axis of the base frame. The configuration in Fig 11(a) has the smallest value and hence the highest stiffness. This configuration could be chosen where accurate position control is required in the x-direction. Conversely, Fig 11(h) has the largest value and hence the largest compliance. This configuration could be chosen for tasks that require force control, robustness to uncertainty, or the ability to absorb impacts in the x-direction.

### Real-Time optimisation in a redundant robot

A kinematic chain, such as a human or robot arm, is redundant if there are more joints than needed to perform a given task. The surplus joints can be used to reconfigure the arm. Mathematically, for a given Jacobian $\text{J}\in R^{\text{m}\times \text{n}}$ it must have full row-rank that is less than the number of joints: rank(**J**) = m < n. If we take the time derivative of Eq 3 we get:

$$\begin{array}{c} \ddot{\text{x}}=\text{J}\left(\text{q}\right)\ddot{\text{q}}+\dot{\text{J}}\left(\text{q},\dot{\text{q}}\right)\dot{\text{q}}. \end{array}$$ (39)

In a redundant system there are infinite combinations of $\ddot{\text{q}}$ to satisfy $\ddot{\text{x}}$. It is therefore possible to reconfigure the arm without moving the endpoint: $\exists \ddot{\text{q}}\neq 0:\text{J}\ddot{\text{q}}=0$.

The dynamic joint torque for controlling the arm is:

$$\begin{array}{c} \tau =\text{M}\left(\text{q}\right)\ddot{\text{q}}+\text{h}\left(\text{q},\dot{\text{q}}\right) \end{array}$$ (40)

where $\text{M}(\text{q})\in R^{\text{n}\times \text{n}}$ is the inertia matrix, and $h(\text{q},\dot{\text{q}})\in R^{\text{n}}$ is a vector of Coriolis and gravitational torques. We can resolve Eqs 39 and 40 by using Gauss’ principle of least constraint [38]. Suppose $\tau_{⌀}\in R^{\text{n}}$ are the joint torques for reconfiguring the arm. We can solve a constrained optimisation problem of the form:

$$\begin{array}{c} \underset{\ddot{\text{q}}}{\text{min}}\;\frac{1}{2}(\tau_{⌀}-\text{M}\ddot{\text{q}}{)}^{\text{T}}{\text{M}}^{-1}(\tau_{⌀}-\text{M}\ddot{\text{q}}) \end{array}$$ (41a)

$$\begin{array}{c} \text{subject}\;\text{to:}\;\text{J}\ddot{\text{q}}=\ddot{\text{x}}-\dot{\text{J}}\dot{\text{q}}. \end{array}$$ (41b)

Using Lagrange multipliers, the solution is:

$$\begin{array}{c} \text{M}\ddot{\text{q}}={\text{J}}^{\text{T}}\underset{\Lambda }{\underset{︸}{{\left({\text{JM}}^{-1}{\text{J}}^{\text{T}}\right)}^{-1}}}(\ddot{\text{x}}-\dot{\text{J}}\dot{\text{q}})+\underset{{\text{N}}_{\text{M}}^{\text{T}}}{\underset{︸}{\left(\text{I}-{\text{J}}^{\text{T}}\Lambda {\text{JM}}^{-1}\right)}}\tau_{⌀} \end{array}$$ (42)

where $\Lambda \in R^{\text{m}\times \text{m}}$ is the apparent inertia of the robot in Cartesian space at the endpoint, and **N**_M_ is the null space projection matrix. Equation Eq 42 can then be substituted in to Eq 40 to control the robot. The operation ${\text{JM}}^{-1}{\text{N}}_{\text{M}}^{\text{T}}=0$ so the task $\tau_{⌀}$ will not produce motion on the endpoint. We can denote the redundant torque vector as:

$$\begin{array}{c} \tau_{⌀}=\alpha \frac{\partial \text{p}}{\partial \text{q}}-{\text{k}}_{\text{d}}\dot{\text{q}} \end{array}$$ (43)

where α∈R is a scalar and k_d_ is a damping factor for stability [39]. By setting the redundant torques proportional to the gradient of Eq 13 the arm will move toward an optimal configuration. Setting *α* < 0 will minimize deflections for a given Δ**x** and maximise stiffness. Conversely, *α* > 0 will maximise compliance. We can rearrange Eq 7 for **w** = **0** to obtain:

$$\begin{array}{c} \ddot{\text{x}}={\ddot{\text{x}}}_{\text{d}}+\Lambda^{-1}(\text{D}\dot{\text{e}}+\text{K}\dot{\text{e}}). \end{array}$$ (44)

This can be substituted in to Eq 42 to control the endpoint. We note that this method of stiffness optimisation has been applied previously in literature (e.g. [8, 23]). The purpose is to illustrate that the resulting arm configurations conform with optimality conditions derived earlier.

#### Results on a planar robot

For the first demonstration of kinematic redundancy we consider simulation of a 4-link mechanism that operates in a 2D plane. The primary task is to hold a fixed position for the end point for m = 2. Since the mechanism has n = 4 joints this gives n − m = 2 degrees of redundancy for it to reconfigure itself. The robot is made to maximize either stiffness or compliance in three different directions:

-
$\hat{\text{x}}=[\begin{array}{cc} 1 & 0 \end{array}{]}^{\text{T}}$ for the x direction,
-
$\hat{\text{x}}=[\begin{array}{cc} 0 & 1 \end{array}{]}^{\text{T}}$ for the y direction, and
-
$\hat{\text{x}}=[\begin{array}{cc} \sqrt{2} & \sqrt{2} \end{array}{]}^{\text{T}}$ for the xy-direction.

**Fig 12:**
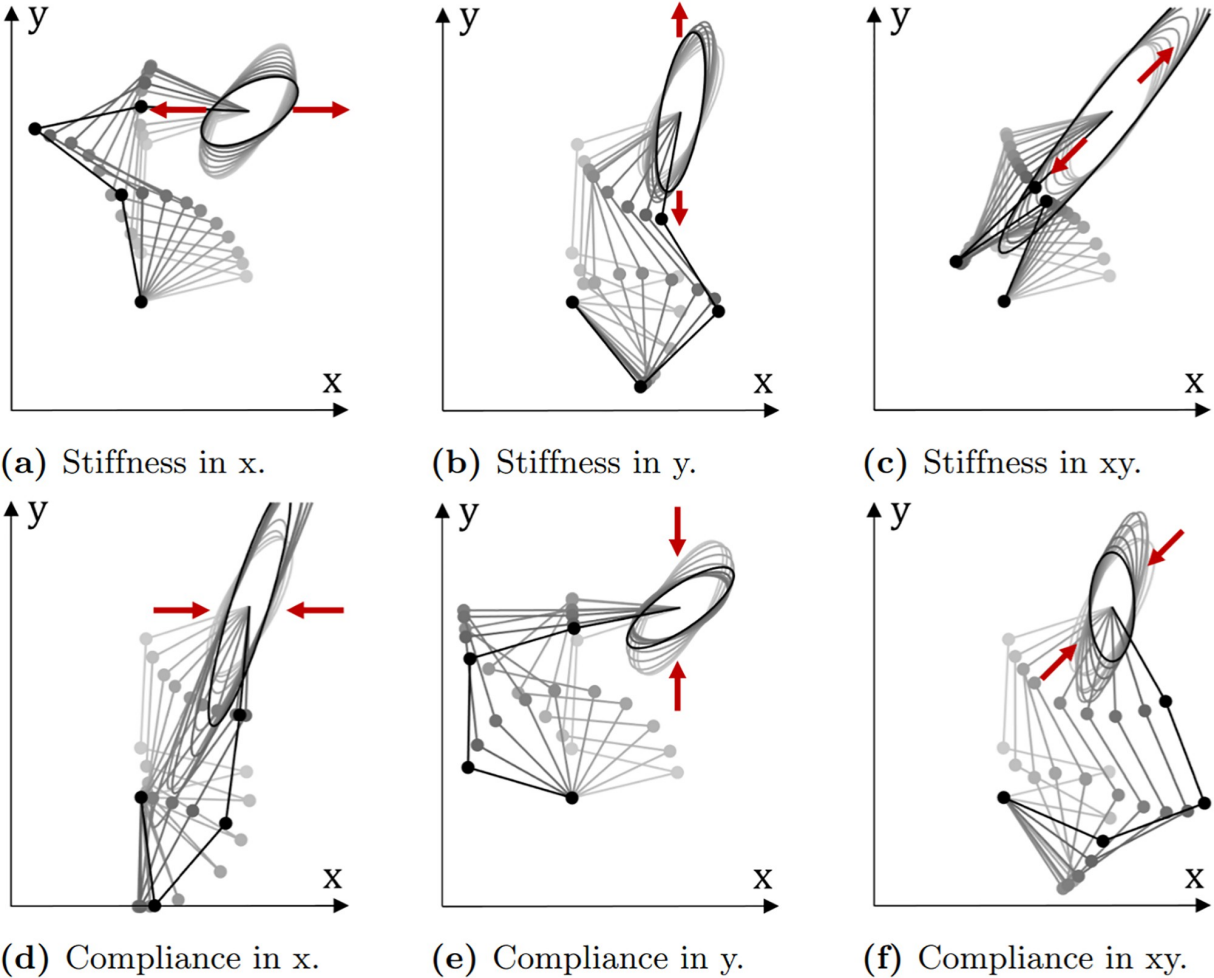
Rotoscoped images of the Cartesian stiffness ellipsoid with changing arm configuration. A robot can be made to increase stiffness or compliance in different directions. Each case begins from the same starting configuration.

Fig 12 shows the changes in the stiffness ellipsoid and arm configuration when applying Eqs 40, 42 and 43. In each of the 6 cases, the robot starts from the same starting configuration. The length of the stiffness ellipsoid either expands or contracts as desired. The final configuration achieved also follows the optimal conditions derived in earlier. For example, in Fig 12(a) the 3rd and 4th links are roughly parallel to the x-axis increasing the stiffness ellipsoid in the required direction. In Fig 12(c) all four links are roughly parallel with the vector $[\begin{array}{cc} \sqrt{2} & \sqrt{2} \end{array}{]}^{\text{T}}$ leading to a larger increase in the stiffness ellipsoid in this direction. These observations are consistent with the optimal stiffness condition Eq 22. For compliance we can see in Fig 12(d) that links 1 to 4 are roughly orthogonal to the x-axis. Likewise, in Fig 12(f), links 1, 3, and 4 are approximately orthogonal to the x-y direction. This has led to a decrease in the width of the Cartesian stiffness ellipsoid in the respective directions. This is consistent with the conditions derived in Eq 33. Of course, the ability to satisfy these conditions fully is constrained by the desired pose for the endpoint and the kinematics of the robot.

#### Emulating human behaviour on a humanoid robot

As mentioned in the introduction, there are many scenarios in which robotics engineers have looked to human motion control for inspiration and insight. A pertinent motive, particularly with humanoid robots, is to instill natural motion that fosters empathy with human collaborators. In this section we apply the redundancy resolution to the ergoCub robot from the Italian Institute of Technology [40]. We emulate the experiments conducted with humans in [12] to show that the control method results in similar arm postures. These results, in conjunction with observations from neurophysiology, suggest that humans are optimising for an underlying physical principle during manipulation tasks. Moreover, by applying these methods to robots we can achieve control that not only appears natural, but enhances task performance.

**Fig 13:**
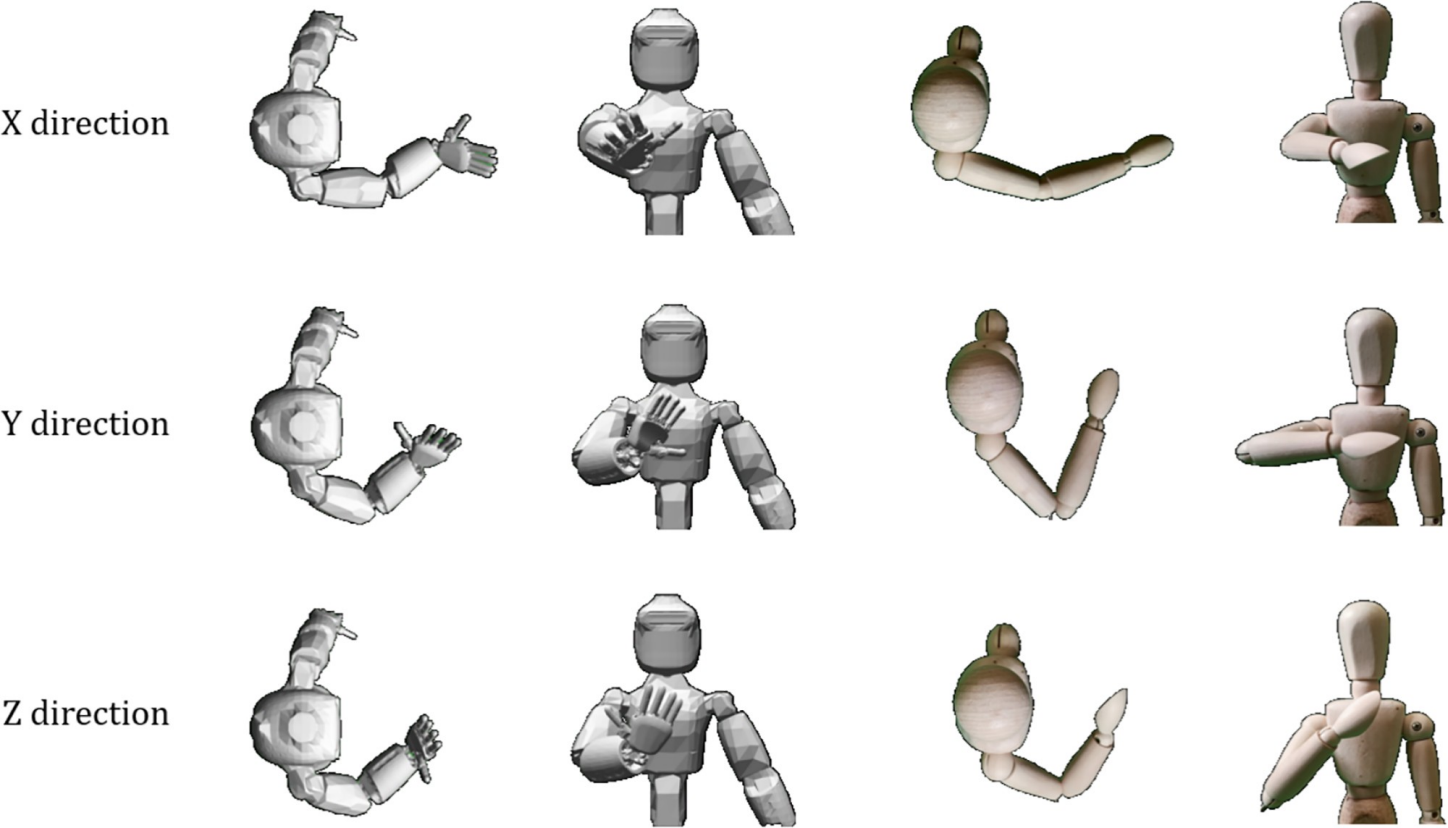
Comparison of a humanoid robot to human experiments. Applying the optimisation principle to a humanoid robot (left) results in similar arm configurations as observed in human.

The primary control task is to keep the right hand at chest height, and constraining it to move along a straight line. The Cartesian stiffness ellipsoid is then maximised in the different Cardinal directions forward (X), sideways (Y), and upward (Z). Fig 13 shows pictures of the final configuration of the ergoCub compared to those observed in humans in [12]. By applying mathematical optimisation of the Cartesian stiffness to a redundant humanoid robot we have achieved similar arm configurations to humans.

The discrepancies between the human and ergoCub are explained by the robot’s joint limits. For example, the elbow has an upper joint limit of 75°. This means the minimum possible angle between the upper arm and forearm is 180° − 75° = 105°. One can see that, for the Y-direction and Z-direction in Fig 13, the ergoCub is unable to bend its elbow further and bring its hand closer to its body the same as a human. Other possible deviations may come from the different proportions of the limbs, and the exact location of the endpoint used for the human experiments.

## Discussion

### An alternative formulation for stiffness estimation

A method for estimating endpoint stiffness in human arms was proposed in [29]. This was later used to estimate stiffness parameters from videos of human manipulation tasks [30]. It relied on the fact that the human arm can be modelled as a 2-link mechanism lying on a plane. If we simply apply the force ellipsoid Eq 5b and use the definition for the column vector Eq 9 to expand it we obtain:

$$\begin{array}{c} {\text{JJ}}^{\text{T}}=[\begin{array}{cc} \sum_{\text{i}=1}^{\text{n}}({\hat{\text{a}}}_{\text{i}}\times {\text{r}}_{\text{i}})({\hat{\text{a}}}_{\text{i}}\times {\text{r}}_{\text{i}}{)}^{\text{T}} & \sum_{\text{i}=1}^{\text{n}}({\hat{\text{a}}}_{\text{i}}\times {\text{r}}_{\text{i}}){\hat{\text{a}}}_{\text{i}}^{\text{T}}\\ \sum_{\text{i}=1}^{\text{n}}{\hat{\text{a}}}_{\text{i}}({\hat{\text{a}}}_{\text{i}}\times {\text{r}}_{\text{i}}{)}^{\text{T}} & \sum_{\text{i}=1}^{\text{n}}{\hat{\text{a}}}_{\text{i}}{\hat{\text{a}}}_{\text{i}}^{\text{T}} \end{array}] \end{array}$$ (45)

The translational stiffness component is given by the sum of outer products:

$$\begin{array}{c} {\text{K}}_{\text{trans}.}=\sum_{\text{i}=1}^{\text{n}}({\hat{\text{a}}}_{\text{i}}\times {\text{r}}_{\text{i}})({\hat{\text{a}}}_{\text{i}}\times {\text{r}}_{\text{i}}{)}^{\text{T}}. \end{array}$$ (46)

From the results of this paper we also know that optimal stiffness and compliance for a (revolute) joint is determined by the cross product $\hat{\text{a}}\times \text{r}$ Eqs 22 and 33. If the arm is in an optimally stiff configuration for a given force vector **f** then the result would be:

$$\begin{array}{c} {\text{K}}_{\text{trans}.}\text{f}=(\sum_{\text{i}=1}^{\text{n}}({\hat{\text{a}}}_{\text{i}}\times {\text{r}}_{\text{i}})({\hat{\text{a}}}_{\text{i}}\times {\text{r}}_{\text{i}}{)}^{\text{T}})\text{f}=0. \end{array}$$ (47)

Therefore, using Eq 45 might be a simpler and more direct method estimating stiffness. Not only can it be applied to any generic serial link mechanism, but it is also possible to obtain the rotational stiffness which is lacking in [29, 30]:

$$\begin{array}{c} {\text{K}}_{\text{rot}.}=\sum_{\text{i}=1}^{\text{n}}={\hat{\text{a}}}_{\text{i}}{\hat{\text{a}}}_{\text{i}}^{\text{T}}. \end{array}$$ (48)

The arm has maximum rotational stiffness for a given moment **m** when:

$$\begin{array}{c} {\text{K}}_{\text{rot}.}\text{m}=(\sum_{\text{i}=1}^{\text{n}}={\hat{\text{a}}}_{\text{i}}{\hat{\text{a}}}_{\text{i}}^{\text{T}})\text{m}=0 \end{array}$$ (49)

which conforms to the conditions in Eq 22.

### Deflection or work?

In this paper we proposed that the magnitude of deflection as the performance criterion for stiffness Eq 13. An alternative may be to consider the work done to/by the endpoint of the arm instead:

$$\begin{array}{c} \text{E}=\frac{1}{2}\delta {\text{x}}^{\text{T}}{\text{K}}_{\text{c}}\delta \text{x}=\frac{1}{2}\delta {\text{q}}^{\text{T}}\underset{{\text{K}}_{\text{a}}}{\underset{︸}{{\text{J}}^{\text{T}}{\text{K}}_{\text{c}}\text{J}}}\delta \text{q}. \end{array}$$ (50)

As with Eq 13, it is evident the work done is a minimum when the Jacobian is singular: **J**^*T*^**K**_*c*_**J**δ**q** = **J**^*T*^**w** = **0**. If humans are attempting to minimise metabolic cost by maximising stiffness, then this would give a direct measure for energy expense. Therefore, future work may be to derive the conditions for optimal stiffness and compliance through this work/energy framing.

### Asymmetry of stiffness

An interesting observation in the simulation results for the planar robot is that optimising for stiffness along one axis does not necessarily equate to compliance in orthogonal axes. In 2 dimensions, the Cartesian stiffness matrix is:

$$\begin{array}{c} {\text{K}}_{\text{c}}=[\begin{array}{cc} {\text{k}}_{x} & {\text{k}}_{\text{xy}}\\ {\text{k}}_{\text{xy}} & {\text{k}}_{y} \end{array}] \end{array}$$ (51)

and its inverse is:

$$\begin{array}{c} {\text{K}}_{\text{c}}^{-1}=[\begin{array}{cc} \frac{{\text{k}}_{y}}{{\text{k}}_{x}{\text{k}}_{y}-{\text{k}}_{\text{xy}}^{2}} & -{\text{k}}_{\text{xy}}\\ -{\text{k}}_{\text{xy}} & \frac{{\text{k}}_{x}}{{\text{k}}_{x}{\text{k}}_{y}-{\text{k}}_{\text{xy}}^{2}} \end{array}]. \end{array}$$ (52)

**Fig 14:**
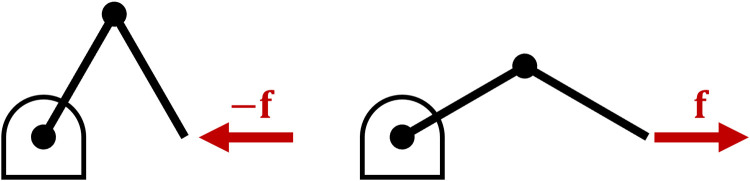
Asymmetry. A serial link mechanism becomes more compliant when pushed in one direction (left), and more stiff when pulled in the other (right).

Compliance in the y-direction is proportional to the stiffness in the x-direction, and vice versa. Yet, if we contrast Fig 12(a) with Fig 12(e) we can see that stiffness along the x-axis has led to a different configuration compared to compliance along the y-axis. For a single rigid body, the stiffness matrix is assumed symmetric for infinitesimally small displacements: ${\text{K}}_{\text{c}}={\text{K}}_{\text{c}}^{\text{T}}$. But for a serial-link mechanism, the joint stiffness Eq 6 is *asymmetric* since ${\text{K}}_{\text{p}}\neq {\text{K}}_{\text{p}}^{\text{T}}$. Consider that if we push on the endpoint of a serial link mechanism (Fig 14), it becomes more compliant as its joint bend in accordance with Eq 33. Conversely, if we pull on the endpoint its stiffness increases as the arm extends as per Eq 22.

This illustrates that Cartesian stiffness Eq 1 is inadequate for studying serial link mechanisms such as a human or robot arm. It is necessary to understand how the joints contribute to stiffness and compliance at the endpoint using Eqs 22 and 33.

## Conclusion

In this paper we have treated Cartesian stiffness control for human and robot arms as a mathematical optimisation problem. We then derived the conditions for the optimal stiffness and compliance with respect to the joints and arm configuration. These optimality conditions explain observations made about human behaviour in the neurophysiology literature. Moreover, optimising this mathematical function in a humanoid robot results in similar configurations to those seen in humans. This suggests there is an underlying physical principle for stiffness control in humans. By understanding the underlying physical principles of human motor control it is possible to embed natural behaviours in to robotics.

## Supporting information

S1 Appendix Proof of inequality between the active and passive stiffness matrices. (PDF)
