# Supplementary material for: Optimal configurations for stiffness and compliance in human & robot arms
Source: PLoS One. 2024 May 29;19(5):e0302987. doi: 10.1371/journal.pone.0302987 (PMC11135727; doi:10.1371/journal.pone.0302987)
Supplement: S1 Appendix — (PDF) [file pone.0302987.s001.pdf]

S1 Appendix. Proof of Inequality between the Active and Passive Stiffness Matrices Assume from Eq 27 that we can write:

$$\mathbf{K}_\mathrm{a} = -\mathbf{K}_\mathrm{p} \tag{53a}$$

$$\mathbf{J}^\mathrm{T}\mathbf{K}_\mathrm{c}\mathbf{J} = -\frac{\partial \mathbf{J}}{\partial \mathbf{q}}^\mathrm{T}\mathbf{w}. \tag{53b}$$

However it can be shown through proof by contradiction that this equality is impossible. First consider that the work done on the endpoint is:

$$\mathrm{W} = \tfrac{1}{2}\delta\mathbf{x}^\mathrm{T}\mathbf{K}_\mathrm{c}\delta\mathbf{x} = \tfrac{1}{2}\delta\mathbf{q}^\mathrm{T}\mathbf{J}^\mathrm{T}\mathbf{K}_\mathrm{c}\mathbf{J}\delta\mathbf{q} \ \geq 0 \ \forall \delta\mathbf{q} \tag{54}$$

where the relationship $\delta\mathbf{x} = \mathbf{J}\delta\mathbf{q}$ has been used Eq 3. Under this condition $\mathbf{J}^\mathrm{T}\mathbf{K}_\mathrm{c}\mathbf{J}$ is at least positive semi-definite. It follows from Eq 53b that $\frac{\partial \mathbf{J}}{\partial \mathbf{q}}^\mathrm{T}\mathbf{w}$ is negative semi-definite and, by extension, symmetric. Expanding this matrix we have:

$$\frac{\partial \mathbf{J}}{\partial \mathbf{q}}^\mathrm{T}\mathbf{w} = \begin{bmatrix} \frac{\partial \mathbf{J}_1}{\partial \mathrm{q}_1}^\mathrm{T}\mathbf{w} & \cdots & \frac{\partial \mathbf{J}_1}{\partial \mathrm{q}_\mathrm{n}}^\mathrm{T}\mathbf{w} \\ \vdots & \ddots & \vdots \\ \frac{\partial \mathbf{J}_\mathrm{n}}{\partial \mathrm{q}_1}^\mathrm{T}\mathbf{w} & \cdots & \frac{\partial \mathbf{J}_\mathrm{n}}{\partial \mathrm{q}_\mathrm{n}}^\mathrm{T}\mathbf{w} \end{bmatrix} \in \mathbb{R}^{\mathrm{n}\times\mathrm{n}} \tag{55}$$

Then from symmetry it must be that:

$$\frac{\partial \mathbf{J}_\mathrm{i}}{\partial \mathrm{q}_\mathrm{j}}^\mathrm{T}\mathbf{w} = \mathbf{w}^\mathrm{T}\frac{\partial \mathbf{J}_\mathrm{j}}{\mathrm{q}_\mathrm{i}} \tag{56a}$$

$$\frac{\partial \mathbf{J}_\mathrm{i}}{\partial \mathrm{q}_\mathrm{j}} = \frac{\partial \mathbf{J}_\mathrm{j}}{\partial \mathrm{q}_\mathrm{i}}. \tag{56b}$$

But we know that this is not true Eq 11. Thus, $\frac{\partial \mathbf{J}}{\partial \mathbf{q}}^\mathrm{T}\mathbf{w}$ is not symmetric and $\mathbf{K}_\mathrm{a} = -\mathbf{K}_\mathrm{p}$ cannot be a solution to Eq 27. ■
